# Severe cardiomyopathy in a young patient with complete deficiency of adipose triglyceride lipase due to a novel mutation in *PNPLA2* gene

**DOI:** 10.1016/j.ijcard.2016.01.137

**Published:** 2016-03-15

**Authors:** Maria Barbara Pasanisi, Sara Missaglia, Denise Cassandrini, Franco Salerno, Stefania Farina, Daniele Andreini, Piergiuseppe Agostoni, Lucia Morandi, Marina Mora, Daniela Tavian

**Affiliations:** a Neuromuscular Diseases and Neuroimmunology, Fondazione IRCCS Istituto Neurologico “Carlo Besta”, Milan, Italy; b Laboratory of Cellular Biochemistry and Molecular Biology, CRIBENS, Catholic University of the Sacred Heart, Milan, Italy; c Molecular Medicine, IRCCS Stella Maris, Pisa, Italy; d Centro Cardiologico Monzino, IRCCS, Milan, Italy; e Departement of Clinical and Community Sciences, Univerisity of Milan, Milan, Italy

**Keywords:** Neutral lipid storage disease, Myopathy, Cardiomyopathy, Triacylglycerol, Lipid droplet, PNPLA2/ATGL

Mutations in the adipose triglyceride lipase (ATGL), an enzyme that hydrolyzes fatty acids from triacylglycerol (TG) stored in multiple tissues into cytoplasmic lipid droplets (LDs), causes the autosomal recessive disorder Neutral Lipid Storage Disease with myopathy (NLSDM) [2]. ATGL protein is coded by *PNPLA2* gene. NLSDM patients are mainly affected by progressive myopathy, cardiomyopathy and hepatomegaly. Their clinical severity appears to be highly variable with particular reference to cardiac involvement [1], [6], [7], [8]. Cardiomyopathy was reported in 36% of the patients. In some of them, it was lethal (25%), or manifested with severe heart condition (31%). Patients with cardiac involvement have adult-onset progressive heart failure, mimicking dilated or hypertrophic cardiomyopathy. The analyses of explanted and autopsied hearts revealed that the defective intracellular hydrolysis of TG results in congestive heart failure with cardiomyocyte steatosis as well as a novel type of coronary atherosclerosis with TG-deposit smooth muscle cells [3], [4], [5]. Molecular mechanisms underlying pathophysiology of heart damage are unknown; moreover, no therapy is available for NLSDM patients.

We report the case of a 26 year-old male patient affected by neutral lipid storage myopathy with severe cardiac involvement. Patient parents were first cousins; a brother died at 3 years of age, during surgery. They referred that the child had always walked on toes, but he never presented weakness or difficulties in physical activity, compared to peers. The patient was first evaluated when he was 11 years-old and was reported to walk on toes, with difficulty to walk on heels, and to have mild calves hypertrophy and reduced tendon reflexes. Blood test revealed high values of CK (1657 U/L), while total and free carnitine levels were normal. Electromyography was normal; an effort test revealed excessive increase in lactic acid levels. He underwent a muscle biopsy that showed abnormal lipid storage. He was diagnosed to suffer from a lipid storage myopathy and therapy with riboflavin was started with some benefit to the patient.

At 20 years of age, the patient complained of walking difficulties and of exercise intolerance. CK levels remained very high (> 2000 U/L). On neurological evaluation he revealed normal strength, mild fatigability, reduced tendon reflexes, walking on forefoot and impossibility to walk on heels. He performed squats without limitation, but with pain at lower limbs. The analysis of plasma free and esterified carnitine resulted normal. A muscle CT detected atrophy and fat infiltration of lower limb muscles. A muscle biopsy revealed numerous vacuoles (Fig. 1A) positive to lipid staining in most fibers (Fig. 1B). Respiratory chain complexes showed normal activity. Electron microscopy showed numerous lipid droplets between myofibrils in most fibers, often causing sarcomere disorganization; while mitochondria were normal (Fig 1C). A neutral lipid storage myopathy was hypothesized and molecular analysis of the *PNPLA2* gene revealed a homozygous novel deletion of seven nucleotides in exon 2 (c.41_47delGCTGCGG) (Fig 1H). The mutated protein was predicted to consist of only fourteen amino acids (p.G14Afs75X). This mutation was submitted to GenBank (accession number KU057409).

During most recent years clinical features of the patient have progressively deteriorated with mild involvement of upper limbs and worsening of distal lower limb weakness; moreover walking difficulties become more evident. Whole-body MRI detected mild fatty infiltration of bilateral trapezius, deltoid and infraspinatus; severe fatty degeneration of pelvic girdle muscles, hamstring, semimembranosus and semitendinosus; diffuse fatty infiltration of leg muscles with partial saving of anterior and posterior tibialis (Fig 1D–G).

The patient has undergone cardiological evaluation at 24 years of age. Cardiac MRI detected left ventricular dilation with diffused hypokinesia, moderate–severe reduction of left ventricular systolic function (ejection fraction FE 36%) and fibrosis of cardiac wall, without fatty infiltration (Fig. 2). A CT excluded coronary pathology, and no innervation defects were seen by myocardial scintigraphy. Holter-ECG revealed sinus rhythm without arrhythmia or conduction defects. A cardiopulmonary exercise test showed a severe reduction in functional capacity mainly due to cardiogenic and extraction defect. After those exams, the patient started a therapy with enalapril 5 mg bid and ivabradine 7.5 mg/die. During the subsequent follow up both exercise capacity and echocardiography ejection fraction improved significantly, indeed oxygen consumption increased from 48% to 57% of the predicted value and a recent echocardiography, performed a year and a half after starting treatment, confirmed the reduction in left ventricle volume and detected a recovery of ventricular function (FE 53%). No indication to implantable-cardioverter-defibrillator (ICD) implantation has been given by the cardiologist so far.

To the best of our knowledge, 44 NLSDM patients have been clinically and genetically characterized until now [3], [6]; about one third of them presented cardiac myopathy. More than 90% of NLSDM patients (with cardiac involvement) harbor highly deleterious *PNPLA2* mutations, causing no ATGL protein production or non-functional ATGL truncated proteins. By contrast, patients with missense mutations – partially preserving lipase activity – have been generally described without cardiac involvement [8]. The *PNPLA2* mutation identified in our patient, determining total loss of ATGL protein, caused early onset and severe myocardial damage. Noteworthy, the patient showed an improvement of cardiac function after therapy with enalapril and ivabradine, in spite of the severe molecular defect. These drugs did not counteract neutral lipid accumulation and yet determined a significant recovery of ventricular function. Informed consent for genetic and clinical analyses were obtained from the patient. Moreover, written informed consent was obtained for publication of this Case report and any accompanying images.

It has already been suggested that molecular and functional analysis of ATGL may improve NLSDM prognosis, paving the way towards personalized therapy. Nevertheless, intra-familial distinct cardiac phenotype has been recently reported in NLSDM [3], underlining that molecular mechanisms regulating the disease development could be influenced by other factors in addition to genotype. Since ATGL deficiency is a very rare disease, the international registry for NLSDM has been established (www.tgcv.org/r/home.html) with the purpose to compare different clinical phenotypes and elucidate NLSDM pathophysiology.

## Funding

This work was supported by grants from GGP14066 from Telethon. The authors gratefully acknowledge EuroBioBank and the Telethon Network of Genetic Biobanks (GTB12001F), for providing biological samples.

## Conflict of interest

None declared.

## Figures and Tables

**Fig. 1:**
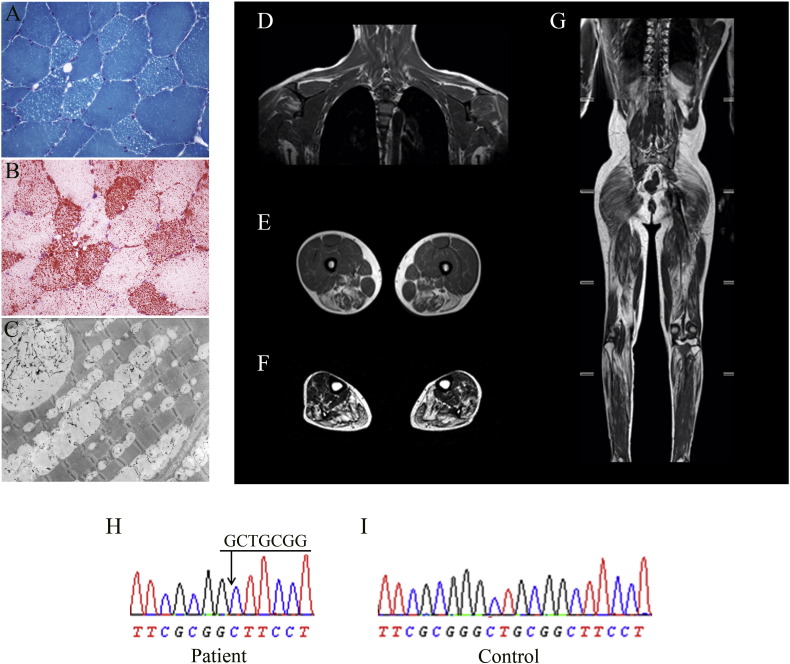
Histochemical, clinical and molecular characterization of NLSDM patient. Light microscopy of frozen transverse sections of skeletal muscle biopsy stained with Gomori trichrome (A) and Oil Red O (B) show an increase of lipid droplets; electron microscopy reveals massive line-up of lipid droplets without signs of mitochondrial alteration (C).Whole-body MRI showing: in (D) mild fatty infiltration of trapezius, deltoid and infraspinatus muscles; in (E) the posterior thigh muscles (hamstring, semimembranosus and semitendinosus) more severely involved than the quadriceps; in (F)severe fatty infiltration of leg muscles, with partial saving of anterior and posterior tibialis; and in (G) the diffuse involvement of the gluteus muscles. Electropherogram of PNPLA2 exon 2 sequence with the c.41_47delGCTGCGG homozygous mutation in patient (H); wild type sequence of exon 2 in a control subject in (I).

**Fig. 2:**
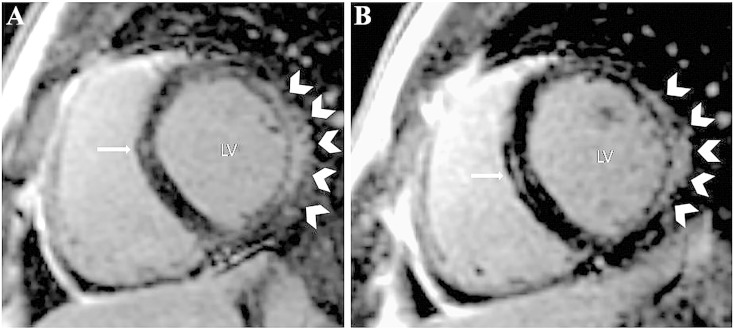
Left ventricle fibrosis detected by cardiovascular magnetic resonance. Contrast-enhanced inversion-recovery gradient-echo images of left ventricle (basal portion, panel A and middle portion, panel B) in short-axis views, showing subepicardial late gadolinium enhancement of interventricular septum (arrows) and meso-subepicardial late gadolinium enhancement of anterior-lateral free wall (head arrows). LV = left ventricle.
